# Contemporary approach to understand and manage COVID-19-related arrhythmia

**DOI:** 10.1186/s43044-021-00201-5

**Published:** 2021-08-30

**Authors:** Omnia Azmy Nabeh, Maiada Mohamed Helaly, Rahma Menshawey, Esraa Menshawey, Mohammed Mansoor Matooq Nasser, Ahmed Mohamed Diaa El-deen

**Affiliations:** 1 Department of Medical Pharmacology, Kasr Alainy Faculty of Medicine, Cairo University, Cairo, Egypt; 2 Kasr Alainy Faculty of Medicine, Cairo University, Cairo, Egypt

**Keywords:** Arrhythmia, COVID-19, Hypoxia, Myocarditis, Adrenergic blockers, Trimetazidine, Ranolazine, ACEIs, ARBs, Troponin, Electrolytes, QTc

## Abstract

Arrhythmia, one of the most common complications of COVID-19, was reported in nearly one-third of diagnosed COVID-19 patients, with higher prevalence rate among ICU admitted patients. The underlying etiology for arrhythmia in these cases are mostly multifactorial as those patients may suffer from one or more of the following predisposing mechanisms; catecholamine surge, hypoxia, myocarditis, cytokine storm, QTc prolongation, electrolyte disturbance, and pro-arrhythmic drugs usage. Obviously, the risk for arrhythmia and the associated lethal outcome would rise dramatically among patients with preexisting cardiac disease such as myocardial ischemia, heart failure, cardiomyopathy, and hereditary arrhythmias. Considering all of these variables, the management strategy of COVID-19 patients should expand from managing a viral infection and related host immune response to include the prevention of predictable causes for arrhythmia. This may necessitate the need to investigate the role of some drugs that modulate the pathway of arrhythmia generation. Of these drugs, we discuss the potential role of adrenergic antagonists, trimetazidine, ranolazine, and the debatable angiotensin converting enzyme inhibitors drugs. We also recommend monitoring the level of: unbound free fatty acids, serum electrolytes, troponin, and QTc (even in the absence of apparent pro-arrhythmic drug use) as these may be the only indicators for patients at risk for arrhythmic complications.

## Background

Arrhythmia is one of the most reported complications in COVID-19 patients [1, 2].Yet the underlying pathophysiology is multifactorial and ill-defined [3, 4]. According to the National Health Commission of China, cardiovascular symptoms were the first clinical presentation in some patients instead of respiratory symptoms, with heart damage being reported in 11.8% of COVID-19 deaths [5]. Chronic cardiovascular conditions such as heart failure, acute coronary syndrome, and arrhythmic disease raise the risk of deleterious COVID-19 effects [5].

## COVID-19-related arrhythmia and their possible pathophysiology

Arrhythmia was reported in about 17% of COVID-19 patients with higher rate (44.4%) among ICU admitted patients [1, 2]. Since COVID-19 most commonly presents as a respiratory illness, this results in a lack of attention and confined differentiation of arrhythmia, which may lead to fulminant outcomes [6]. Considering the types of arrhythmias, atrial fibrillation (AF) had the greatest prevalence rate (21%) according to Gopinathannair et al. global study that included 1197 participants from 76 countries. Gopinathannair et al. reported atrial flutter (Aflt) in 3.5% and sustained atrial tachycardia (AT) in 5.7% of admitted patients [7]. Similarly, in a cohort of 69 COVID-19 patients who were admitted in medical ICU, Colon et al. (2021) reported supraventricular arrhythmia (AT, AF and Aflt) in 16.5% of patients [8]. Other studies have reported sinus tachycardia in 1% [9] and ventricular tachycardia (VT)/ ventricular fibrillation (VF) in 5.9% [10]. On the other hand, sinus bradycardia, high degree AV block, and sick sinus syndrome were also reported in some case reports [11, 12].

### Hypoxia

Hypoxia is one of the fulminant complications of COVID-19. Indeed, Xie et al. noted that dyspnea (with SPO_2_ cut off 90% after receiving oxygen aid) was a strong significant predictor of mortality among COVID-19 patients [13]. Among the causes of hypoxia in COVID-19, acute respiratory distress syndrome (ARDS) is one of the predictable muddles embroiling COVID-19 outcomes. It causes marked alveolar damage, interstitial widening with edema, inflammatory cells infiltrates, and a significant decrease in oxygen saturation [14]. Hypoxia also may result from a pulmonary embolism as microvascular thrombosis is well documented to complicate COVID-19 and evidenced by the elevated D-dimer, fibrinogen levels, and subsegmental vascular enlargements in areas showing ground glass appearance. Moreover, precapillary shunts could result from enhanced inflammatory reaction [15]. These shunts will cause unequal distribution of pulmonary capillary flow, with hyperperfusion of inflamed segments and hypoperfusion of normal segments, which in turn results in hypoxia [16]. Furthermore, Tobin et al. [17] have suggested that SARS-COV-2 may get access through the angiotensin converting enzyme 2 (ACE2) that is expressed on the nasal mucosa, to reach the brain via the olfactory bulb and then affects the respiratory centers. Tobin et al. also referred to the possible alteration in chemoreceptors upon SARS-COV-2 infection as ACE2 is also expressed in the carotid bodies and consequently leads to acute respiratory failure and hypoxia.

The interplay between hypoxia and arrhythmia has been studied widely, and the relation between types of hypoxia: acute, intermittent and chronic, and arrhythmia has been confirmed [18]. One of the mechanisms by which the cardiac cells sense hypoxia is the phosphorylation of ion channels or the alteration in the reduction/oxidation (redox) state inside the cell. Redox, through modification of the sulfhydryl groups of cysteine residues impairs many ion channels gating, ion transporters, and enzymatic activity [19]. Ca^2+^, which is a main component of the cardiac excitation–contraction coupling, is a susceptible target for redox deleterious effects through redox-induced alteration of: ryanodine receptors (RyR), Ca^2+^ ATPase, and longlasting Ca^2+^ channels (CA_L_) [20]. Similarly, hypoxia was reported to increase the Ca_L_ sensitivity to β-adrenergic stimulation, which gives rise to pro-arrhythmic after depolarization [21].

Small ubiquitin-like modifier (SUMO) belongs to the ubiquitin-like polypeptides family that can alter the half-life, function, and/or location of different target proteins by forming covalent bonds with them [22]. In fact, acute hypoxia causes rapid SUMOylation of the voltage gated Na^+^ channel; Na_v1.5_, which in turn results in an increased late/persistent Na^+^ current (*I*_PNa_) up to tenfold [23]. On the same context, redox was also reported to increase the persistent Na^+^ current that results in Na-dependent Ca-overload and pro-arrhythmic triggered activity [24].

Moreover, hypoxia (at a level that causes metabolic inhibition) increases ATP-sensitive K^+^ channels through redox modification. These alterations can cause prolonged repolarization and facilitate arrhythmogenic after depolarization [3, 4].

On the other hand, hypoxia through dephosphorylation of connexin 43 in gap junctions could result in electrical uncoupling and tissue anisotrophy [25].

### Catecholamines and arrhythmia

In contrast to chronic hypoxia that decreases the number and the sensitivity of functioning β adrenergic receptors, acute hypoxia causes an enhanced catecholamine secretion through the stimulation of peripheral chemoreceptors and the sympathetic flow [25].

Additionally, SARS-COV-2 infection is associated with hyper-inflammation state that could lead to cytokine storm [26]. Staedtke et al. [27] documented an association between these inflammatory conditions and the catecholamine surge. These cytokines can stimulate the activation of the cardiac sympathetic system through central (hypothalamus-mediated) and peripheral (left stellate ganglia-mediated) pathways [28]. Moreover, ACE2 was found to reinforce the inhibitory signals to the paraventricular neurons (PVN), which regulate sympathetic activity [29]. Thus, it is expected that downregulation of ACE2 after binding to SARS-COV-2 will strengthen the sympathetic output which successively stimulates the renin angiotensin aldosterone system (RAAS) to increase angiotensin II and maintain a close loop of sympathetic activation.

Although catecholamine in physiological levels is essential for immune regulation, supraphysiological catecholamine secretion may result in immune dysregulation that maintains a self-amplifying loop that could lead to significant multi-organ damage and myocardial injury [30], which may give rise to arrhythmias that will be discussed later in the text.

As regards the molecular pathway for arrhythmia, adrenergic stimulation of adenyl cyclase increases cAMP levels, which generates a larger current at the same voltage and moreover enhances RyR2 and Na^+^/Ca^2+^ exchanger (NCX-1) activity [31].

Moreover, Lu et al. [32] and Zhang et al. [33] reported that, during hypoxia, there is an enhanced sympathetic activity with shortening of the effective refractory period (ERF) and a non-uniform reduction in ERP which lead to transmural dispersion of repolarization (TDR) and reenterant arrhythmia.

It is noteworthy that sympathetic over-activity was reported in both AF and Ventricular arrhythmia [34]. Even so, sympathetic surge, in patients with atrioventricular nodal reentrant tachycardia (AVNRT) or AVRT, accelerates and facilitates the conduction of the AF/Aflt through the AVN to depolarize the ventricles and then increases the susceptibility for VF and SCD [35]. Similarly, Rédéric et al. [36] have observed a strong correlation between arterial oxy-hemoglobin desaturation /QTc length and the susceptibility to ventricular arrhythmia influenced by the sympathetic alteration of the rate dependence of ventricular repolarization. Moreover, catecholamines may reverse the action of antiarrhythmic drugs and hence mandate the need for cardiac sympathetic denervation for patients with refractory malignant ventricular arrhythmia [34].

On the other hand, catecholamine surge leads to mobilization of lipids from adipose tissue in the form of free fatty acid (FFA) [37] and impairs pancreatic insulin production and sensitivity [63]. When the FFA level exceeds that of the albumin, it accumulates in the extracellular space as unbound-FFA that can freely cross the cell membrane and accumulate inside the cells. Although FFA serves as the main source of energy to the cardiac myocytes, beta-oxidation of FFA (in hypoxic state) would utilize more oxygen and produce less adenosine triphosphate (ATP).This causes further myocardial ischemia. Besides, the resulting lactic acid will accumulate inside the cardiac myocytes and lower the cellular PH with several hazardous effects on the cell membrane and cellular enzymes that subsequently lead to arrhythmia [38].

### Myocardial injury (myocarditis, cytokine storm), acute coronary syndrome, and arrhythmia

Myocarditis refers to inflammation of the heart without ischemia. The COVID-19-related myocarditis has been documented in many cases. However, the actual prevalence of myocarditis in COVID-19 patients is not yet accurate as many cases were presented with cardiac symptoms and managed accordingly without diagnosis of COVID-19 [39].

Myocarditis can occur by either direct viral damage or secondary to systemic hyperinflammation due to host immune response. Cardiac myocytes express ACE2 receptor through which, SARS-COV2 gains access to enter the cardiac cells where they replicate and damage the cell. Shortly, the antigen presenting cells will deliver the SARS-COV-2 antigen to the Naïve T lymphocytes to turn into cytotoxic CD8^+^ T cells that will migrate to the infected myocytes and cause more inflammation and myocyte damage. In case of cytokine storm, the pro-inflammatory cytokines will activate more cytotoxic T cells that in-turn will release more cytokines and cause more myocyte destruction [40].

Of these cytokines, interleukin (IL)-6 plays a pivotal role in arrhythmia generation as it causes displacement of a desmosomal protein named plakoglobin that is responsible for cell-to-cell adherence and hence it causes cell membrane damage. Both, cell membrane damage and inflammatory edema will disturb electrical conduction and may cause arrhythmia. Other proposed mechanism for arrhythmia in COVID-19-related myocarditis is the re-entrant tachycardia upon myocardial fibrosis or scars. Furthermore, exaggerated inflammatory cytokines especially tumor necrosis factor-alpha (TNF-α) and IL-6, alter the expression and function of cardiac ion channels especially the delayed rapid rectifier K^+^ channels (*I*K_r_) and Ca^2+^ channels which cause prolongation of ventricular action potential (AP) and may predispose to prolonged QTc syndrome and Torsade de points (TdP). IL-6 additionally inhibits cytochrome P450 activity and accordingly can increase the bioavailability of many medications and worsen the drug–drug interactions and incline QTc/TdP arrhythmia [28, 41].

Theoretically, the downregulation of ACE2 (that are saturated with SARS-COV2) leads to accumulation of angiotensin II(AgII) that will cause an unopposed action via AgII type 1 receptors (AT1) and prompt catecholamine release and precipitate arrhythmia. Timmermans [42] delineated that AT1 stimulation by AgII will increase Ca^2+^ influx via Ca_L_ channels and sarcoplasmic reticulum that will cause Ca^2+^ overload and precipitate arrhythmia. In the same way, the AT1 blocker, losarten, was found to successively prevent reperfusion ventricular arrhythmia through abolishing AgII-dependent Ca^2+^ overload [43]. Ag II also through enhancing cardiac fibrosis and cardiac remodeling gives rise to reenterant arrhythmia [44]. Moreover, AgII overactivity increases ROS production that targets: ion sarcolemma ion channels, sarcoplasmic reticulum Ca^2+^-ATPase (SERCA), RyR2 and connexin 43, and so on increase the incidence of arrhythmia in susceptible patients [45]. Similarly, aldosterone increases Ca_L_ expression [46] and downregulates transient outward potassium channels (*I*K_to_) [47] and hence causes AP prolongation.

In the same perspective, there is a close correlation between COVID-19 and cardiac ischemia in susceptible patients. Hyperinflammation together with catecholamine mediated vasoconstriction (in the presence of a preexisting atheromatous plaques and/or vasculitis) could lead to serious coronary occlusion events and myocardial ischemia. Moreover, IL-6 and TNFα can cause depletion in both coagulation and fibrinolytic factors and consequently disseminated intravascular coagulation [3]. Coincidentally, the presence of hypoxia and the enhanced FFA metabolism, as mentioned previously, would lead to more myocardial ischemia and arrhythmia.

It goes without saying that myocardial ischemia will impair cellular metabolism with deleterious effects on the ion channels and ion currents as it increases the intracellular concentrations of Na^+^ and Ca^2+^, increases and decreases extracellular K^+^ and Na^+^, respectively, and decreases the intracellular ATP and pH levels^39^.

On the other hand, despite acute coronary syndrome hospital admissions were expected to rise throughout the COVID-19 pandemic, the number has declined in comparison with last year reports in some countries [48]. Although the actual causes are unknown, this may refer to the impact of physical rest during the quarantine or the fear to seek medical advice (to avoid getting infected with the virus).

### Takotsubo cardiomyopathy

It is well known that Takotsubo cardiomyopathy (TCM) is one of the reported complications of COVID-19 [49, 50]. Although TCM usually runs a benign course, life-threatening arrhythmia (VT, VF, and AV block) has been reported in about 8% of patients and recently has been linked to poor survival rates^89^. Qtc prolongation was accused to cause triggered arrhythmia. However, a considerable number of patients had experienced arrhythmia with normal QTc that have been explained by the presence of myocardial edema and micronecrosis that may result in dispersion of repolarization and electrical heterogeneity. Yet, the malignant impact of catecholamine on arrhythmia generation is more obvious in patients with TCM [50].

### QTc prolongation and arrhythmogenic drugs

With regard to QTc prolongation, one study found that QTc prolongation was present in a proportion of 13 percent of their patients, with a mean QT interval of 431 milliseconds [2].

Drug therapies (such as azithromycin, hydroxychloroquine (HCQ), and lopinavir/ritonavir) used in treatment protocols for COVID-19 infections that have a direct influence on arrhythmogenesis, or by affecting the metabolism of other drugs which have these effects, were implicated in the development of QTc prolongation [51].

HCQ drugs have many well-reported cardiovascular effects, including the increased depolarization length duration, as well as purkinje fiber refractory period, associated with their long term use. These effects ultimately cause AV nodal/His system dysfunction. HCQ is accumulated in within the lysosomes, where it inhibits phospholipase activity. This results in the formation of inclusion body formation, and elevation of lysosomal pH which inactivates proteins; these properties lead to the development of both atrial and ventricular arrhythmias. HCQ itself can induce QT prolongation by an inhibitory effect on Ca_L_ and delayed rectifier potassium currents [52, 53]. Moreover, HCQ can inhibit funny current channels in pacemaker cells, which decreases the heart rate and can also cause AV conduction block [1].

Azithromycin results in prolongation of AP through their inhibition of hERG-K + channels. This, along with unopposed Na^+^ and Ca^2+^ currents, leads to the development of TdP [54].

QT prolongation is dose-dependent and patients taking HCQ with concurrent azithromycin are at greater risk of QT changes and cardiac arrest than either drug used alone [54].

Another way these drugs can cause arrhythmias indirectly due to any associated renal disturbances, such as acute kidney injury, which can result in electrolyte abnormalities. Electrolyte abnormalities among hospitalized patients are well documented and a connection exists between this event and the development of arrhythmias, especially in those with preexisting arrhythmias [55].

Furthermore, hyperferritinemia which is a common clinical presentation of COVID-19 infection [56] may also be linked to the development of QTc prolongation. In accordance with this theory, a study assessing the causes of prolonged cardiac repolarization in 20,261 Danish males, Henriksen et al. (2016) found that elevated iron storage, independent of inflammation or genetic causes (hemochromatosis) was significantly associated with QTc prolongation [57].

Thus, the risk of QTc prolongation, in COVID-19 patients, involves the interaction between many factors, all of which raise the risk for TdP and cardiac arrest.

### Bradyarrhythmia and SCD with COVID

Many case reports and case series reported the association between COVID-19 and bradyarrhythmia. Most of these cases suffered from atrioventricular block and others suffered from sinus node dysfunction. Though Troponin T was mildly elevated in few cases as an indicator of myocarditis, other cases did not show significant Troponin changes. Unfortunately, myocardial MRI was not available to exclude or confirm myocarditis. Nevertheless, this may exclude myocarditic or acute coronary syndrome as the only definitive cause for bradyarrhythmia. However, the affinity of SARS-COV2 to the abundant ACE2 receptors on the cardiac myocyte as well as the presence of the viral RNA on cardiac autopsies from patients died from COVID-19 complication suggest direct myocardial injury and inflammation of the conductive system besides the systemic hyper-inflammation state as a possible underlying pathology [12, 58].

In view of the association of COVID-19 and sudden cardiac death (SCD), Baldi et al. [59] observed that out of 362 SCD victims, 103 were suspected or diagnosed with COVID-19. Similarly a twofold rise in SCD cases was observed in Paris during this SARS-COV2 pandemic [60].

Obviously, considering all the previous pathologies and their impact on the heart, one should have a high index of suspicion with proper close follow-up and apply the adequate preventive measures to reduce SCD.

## Management of COVID-19 infection

**Fig. 1:**
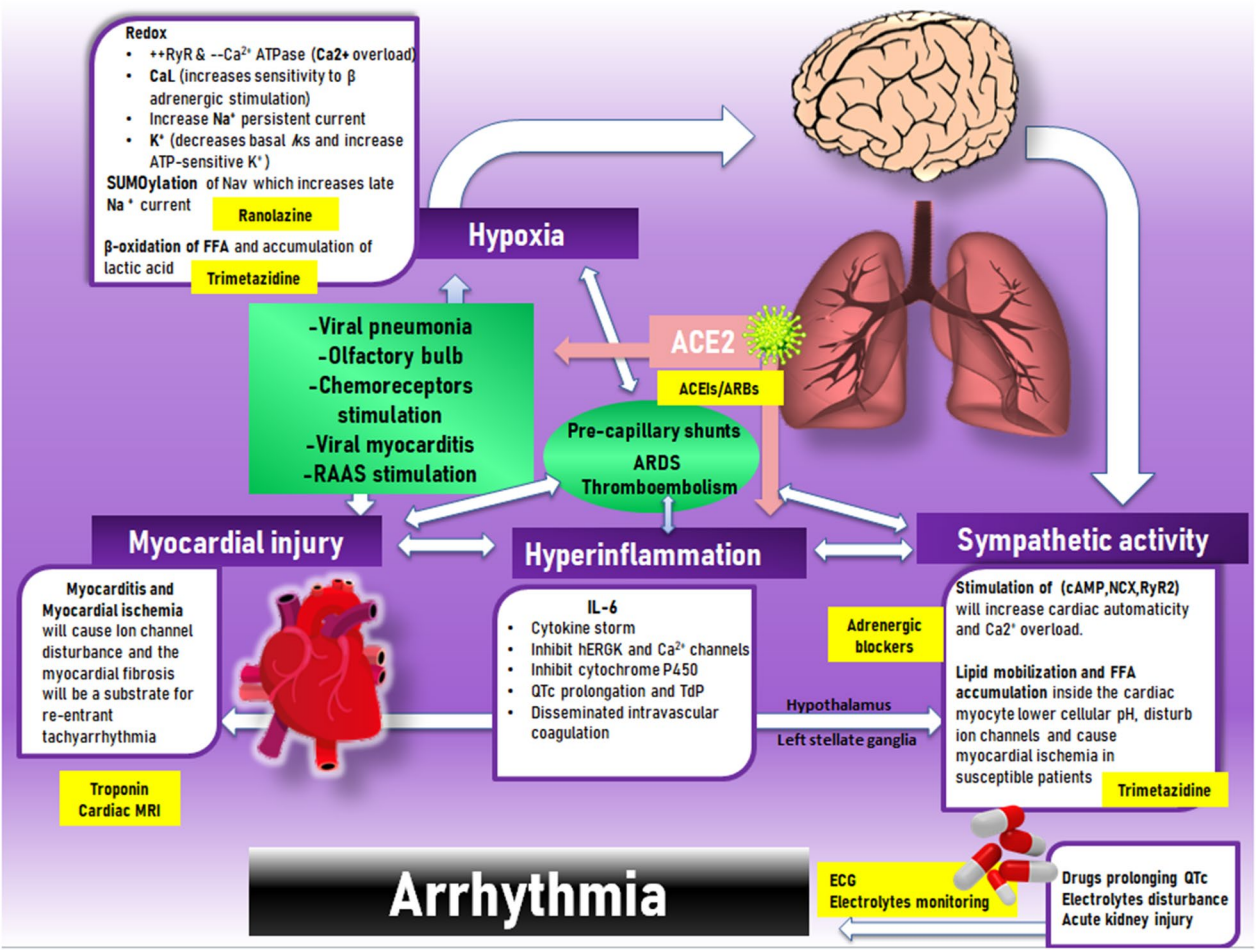
Mechanism of arrhythmia in COVID-19 infection and the proposed management protocol. ACEIs: angiotensin converting enzyme inhibitors; ARBS: angiotensin receptor blockers; ARDS: adult respiratory distress syndrome; ATP: adenosine tri-phosphate; FFA: Free fatty acid; hERG: Human ether-a-go-go-related gene; IL-6: Interleukin-6; MRI: magnetic resonance imaging; Nav: voltage gated Na^+^ channels. RAAS: renin angiotensin aldosterone system; RyR: Ryanoide receptors; Tdp: Torsade de points

Apart from antiviral, antimicrobial, steroidal, and anticoagulant drugs [61], management of COVID-19 patients should include a strategy for prevention, early detection and treatment of COVID-19-related arrhythmia. Here we discuss some drugs that may play an important role in the future to prevent arrhythmia in selected patients (Fig. 1).

### Trimetazidine (TMZ)

Considering the proficiency of both hypoxia and catecholamines to impair the balance between glucose oxidation and glycolysis and to enhance the mobilization and β-oxidation of FFA, that increases oxygen requirement and lowers cellular pH, the antianginal drug, TMZ may have a protective role to guard against arrhythmia.

TMZ, as a cardioprotective drug can inhibit long chain 3-ketoacyl coenzyme A thiolase (LC 3-KAT) which is the final enzyme in the FFA β-oxidation pathway and increases the pyruvate dehydrogenase activity. These result in an increase in glucose metabolism, decreased oxygen consumption during ATP synthesis, decreased hydrogen ion production, limited increase of intracellular acidosis and reduced Ca^2+^ ion accumulation. Through correcting energy insufficiency, TMZ reduces formation of reactive oxygen species (ROS), decreases sodium accumulation inside myocytes, and reduces neutrophil infiltration with subsequent stabilization of cardiomyocyte membranes [62].

In addition to its metabolic effects, Ruixing et al. [63] suggested that inhibiting cell apoptosis could be an additive cardioprotective effect of TMZ, while Liu et al. [64] suggested that TMZ might have protective effects on cardiac fibrosis caused by pressure overload. Also, it was reported that TMZ was suggested to improve endothelial function by preserving endothelial progenitor cells with increasing nitric oxide production. Furthermore, TMZ was associated with improved inflammation in several animal models of sepsis [65].

Owing to these effects, we encourage the clinical assessment of the role of TMZ to prevent hypoxic/sympathogenic arrhythmia in patients with myocardial ischemia with close monitoring of unbound-FFA level as an indicator for vulnerable patients.

### Ranolazine

Similarly, ranolazine (RNZ) though considered an antianginal drug that can potentiate glucose metabolism in ischemic patients, yet it has many antiarrhythmic potentials. As mentioned before, inflammatory cytokines and the late Na^+^ current are encountered in the pathogenesis of COVID-19-related arrhythmia. Interestingly, this antianginal drug has an anti-inflammatory potential through enhancing peroxisome proliferator-activated receptor gamma (PPAR-ɣ) expression and subsequently, ameliorating the transcription factor; nuclear factor kappa ligand B (NFκ B) mediated cytokine production: IL-1β and TNFα [66].

Furthermore, RNZ inhibits *I*Na_late_ that decreases the forward activity of NCX-1 and eventually decreases the cardiac myocytes Ca [2]^+^ load and hence prevents triggered arrhythmia. Yet, RNZ can affect other ion currents (*I*k_s_, *I*k_r_, and *I*_Ca_) that cause a small increase in AP duration with QTc prolongation on surface ECG. However, unlike other pro-arrhythmic drugs, this QTC prolongation with RNZ is not associated with increased risk to TdP, as RNZ reduces TDR and prevent after depolarizations [67].

The antiarrhythmic effects of RNZ were approved in many clinical studies. Murdock et al. [68] reported that RNZ successfully prevented the early relapse of AF. Moreover, RNZ was found to be of complementary effects with other antiarrhythmic drugs; amiodarone [69] and dronedarone [70] in managing AF.

Even so, considering the effect of RNZ on QTc prolongation and being a substrate for P-glycoprotein and cytochrome P450, particularly CYP3A4 and CYP2D6 [70], the use of RNZ is better to be restricted for those patients without preexisting prolonged QTc state and those not receiving QTc prolonging medications.

### Adrenergic antagonist

Beta blockers (B.B) as antiarrhythmic drugs antagonize many arrhythmogenic sympathetic hazards and are one of the corner stones in prevention of arrhythmic death related to myocardial infarction, cardiomyopathy, heart failure [71], and recently viral myocarditis [72]. In addition to their antiarrhythmic potential, beta adrenergic receptor on the alveolar macrophages was accused with the hyper-IL-6 secretion and the resulting inflammatory and prothrombotic state. Moreover, beta blockers were reported to decrease the level of circulating TNFα and IL-6 while increasing IFNɣ [73] and hence beta blockers may play an important immune-regulatory role in the pathogenesis of COVID-19 cytokine storm and dampen the catecholamine surge deleterious effects and consequently prevent arrhythmia.

By the same taken, Vogelstein et al. [74] suggested α_1_-adrenergic antagonists -and not β-adrenergic antagonists- as a therapeutic option to prevent acute respiratory distress syndrome and cytokine storm. Vogelstein based this opinion upon a retrospective study from 2 cohorts of patients admitted to hospitals between (2007 and 2015) who were receiving α_1_-adrenergic antagonists to treat chronic non-respiratory conditions. They observed that α_1_-adrenergic antagonists prevented rather than treated hyper-inflammation in patients at risk to develop cytokine storm. Similarly, Koenecke et al. [75] reported 34% relative risk reduction for death or the need to mechanical ventilation with the use of α1-adrenergic antagonists in patients with lower respiratory tract infection. On the same context Konig et al. [76] concluded that α_1_-adrenergic antagonists may protect from ARDS and cytokine storm.

So, in view of hyperinflammation/arrhythmia relationship, we assume that alpha and beta blockers could be of adjunctive use in naive COVID-19 patients at risk to develop arrhythmia, while are necessary to those with preexisting coronary artery diseases, compensated heart failure and/or myocarditis. The role of adrenergic blockers relies on their ability to: 1. dampen RAAS over-activity and hence decrease the expression of ACE2 and hence viral entry, 2. ameliorate the hazardous effects of catecholamines over-secretion on the inflammatory cytokines, and 3. prevent or treat cardiac arrhythmia. However, the use of adrenergic blockers in the management protocol of COVID-19 is challenging and needs to be figured out case-by-case.

### ACE inhibitors

The use of angiotensin converting enzyme inhibitors (ACEI) and angiotensin receptor blockers (ARBs) in the setting of SARS-COV2 infection is debatable (either beneficial or harmful based on evidence). Currently, there is a theory that the use of ARBs and ACEI increases the risk of developing severe COVID-19 infection. This theory was supported based on the fact that using ARB/ACEI upregulates the expression of ACE2, which is the main mechanism by which SARS-COV-2 enters and infects cells, thereby increasing the infectivity, morbidity, and mortality^135^.

In the same context, Selcuk et al. [77] noted that the use of ARBs and ACEIs had an increased risk of needing ICU admission and endotracheal intubation in COVID-19 patients. However, Flacco et al. [78] in a systematic review and metanalysis regarding the association of using ARBs and ACEIs and the development of severe/lethal COVID-19 infection found contradicting results.

However, as regards arrhythmia, the role of ACEIs and ARBs are still not fully known. Experimental studies have shown that AgII causes dispersion of the cardiac AP duration and are involved in reperfusion arrhythmia. Moreover, past investigations have described that ACEIs and ARBs have antiarrhythmic properties by preventing the electrical and structural remodeling of cardiac cells [78]. Furthermore, in a study be De mello (2002) [79], the ACEI, enalapril, showed enhanced impulse conduction through incrimination of membrane potential and improving cell coupling which may prevent re-entrant arrhythmia and reduced automaticity as well.

Another explanation may be that ACEIs stabilize the electrolyte concentration in certain tissues of the body including the heart, which is highly sensitive to alterations in calcium, potassium, and sodium levels [80].

In a retrospective study assessing whether ACEI and ARBs result in direct current cardioversion of arrhythmias done by Makker et al. [81] found that 96% of the 107 patients with persistent AF achieved normal sinus rhythm after the use of ACEIs. However, clinical trials may be needed to determine the use of ACEIs and ARBs in the setting of COVID-19 induced arrhythmia, which has yet to be studied.

### Colchicine

Colchicine is one of the non-antiarrhythmic drugs that seems to have a potential role in preventing arrhythmia related to myocardial injury and myopericarditis caused by SARS-COV2 invasion. Duran et al. have reported a significant improvement in P wave indices, especially that related to P wave duration, the length of PR interval and the isoelectric interval with colchicine therapy in patients with myopericarditis compared to control group [82]. This can be explained by the potent anti-inflammatory role of colchicine that can prevent neutrophilic activation and cytokine production which sequentially stabilize the cardiac microtubules and ion channel system [83].

From this data, it can be noted that there is still no clear evidence about the COVID-19/arrhythmia relationship. Robust research is necessary in order to determine the exact mechanism by which conductional abnormalities occur in the setting of COVID-19, and how we can use this knowledge to prevent worsening outcomes.

## Conclusions

The pathophysiology of COVID19-related arrhythmia is multifactorial. The evaluation of serum electrolytes, troponin levels, and frequent ECG assessment in all COVID-19 patients, as well as additional cardiac MRI assessment (in case of myocardial injury) and measurement of unbound-FFA level (for ICU patients) could be the only clues to stratify patients with arrhythmic risk. Comprehensive research is necessary in order to determine the exact mechanism by which conductional abnormalities occur in the setting of COVID-19, and the possible beneficial effects of adrenergic blockers, trimetazidine, ranolazine, ACEI, and colchicine.

## Data Availability

Not applicable.

## Abbreviations

ACE: Angiotensin converting enzyme
AF: Atrial fibrillation
Aflt: Atrial flutter
AgII: Angiotensin II
ARB: Angiotensin receptor blocker
ARDS: Acute respiratory distress syndrome
AP: Action potential
AT: Atrial tachycardia
AT1: Angiotensin II type 1 receptor
ATP: Adenosine triphosphate
AVNRT: Atrioventricular nodal reentrant tachycardia
B.B: Beta blockers
Ca_L_: Longlasting Ca^2^^+^ channels
Ca_T_: Transient Ca^2^^+^ channels
CYP: Cytochrome P
ECG: Electrocardiogram
ERP: Effective refractory period
FFA: Free fatty acids
hERG: Human ether-a-go-go-related gene
HCQ: Hydroxyl chloroquine
ICU: Intensive care unit
IFNɣ: Interferon gamma
*I*_PNa_: Late/persistent Na^+^ current
*I*K_r_: Rapid rectifier outward potassium channels
*I*K_s_: Slow rectifier outward potassium channels
*I*K_to_: Transient outward potassium channels
IL: Interleukin
NCX: Na^+^/Ca^2^^+^ exchanger
NFκB: Nuclear factor kappa ligand B
PPAR-ɣ: Peroxisome proliferator-activated receptor gamma
QTc: Corrected QT
RNZ: Ranolazine
RyR: Ryanodine receptor
SCD: Sudden cardiac death
SUMO: Small ubiquitin-like modifier
TCM: Takotsubo cardiomyopathy
TdP: Torsade de points
TDR: Transmural dispersion of repolarization
TMZ: Trimetazidine
TNFα: Tumor necrosis factor alpha
VF: Ventricular fibrillation
VT: Ventricular tachycardia
